# Postoperative Anaemia Severity as a Superior Predictor of Short-Term Adverse Outcomes Following Vascular Surgery: A Retrospective Cohort Study at a UK Teaching Hospital Trust

**DOI:** 10.7759/cureus.113218

**Published:** 2026-07-23

**Authors:** Ka Yee Chaw, Jamie Zhi Guo Ong, Murtaza Karimjee Salem

**Affiliations:** 1 General Medicine, Nottingham University Hospitals NHS Trust, Nottingham, GBR; 2 Vascular Surgery, Nottingham University Hospitals NHS Trust, Nottingham, GBR

**Keywords:** 30-day mortality, 30-day outcomes, 30-day readmissions, anaemia severity, postoperative anaemia, prolonged hospital stay, united kingdom healthcare

## Abstract

Background

Perioperative anaemia is highly prevalent in vascular surgery, yet the independent prognostic significance of postoperative anaemia severity remains poorly characterised compared with preoperative anaemia. This study evaluated the association between postoperative anaemia severity and short-term adverse outcomes and compared its predictive utility with preoperative and persistent perioperative anaemia models.

Methods

A retrospective single-centre cohort study was conducted at Nottingham University Hospitals NHS Trust. All patients undergoing inpatient vascular surgical procedures between July and October 2024 were identified. After excluding day-case procedures and patients with incomplete perioperative haemoglobin (Hb) data, 196 patients were included. Anaemia severity was categorised using World Health Organisation (WHO) sex-specific thresholds into four levels: no anaemia, mild (Hb 110-129 g/L), moderate (Hb 80-109 g/L), and severe (<80 g/L). The primary outcome was a 30-day composite of prolonged hospitalisation (seven days or more), unplanned readmission, and all-cause mortality. Three binomial logistic regression models were compared using Akaike Information Criterion (AIC), Nagelkerke R², and area under the receiver operating characteristic curve (AUC).

Results

Among 196 patients (mean age 69±15.4 years), the 30-day composite adverse outcome occurred in 158 (80.6%), including prolonged hospitalisation in 140 (71.4%), unplanned readmission in 50 (25.5%) and mortality in 16 (8.2%). A clinically significant perioperative haematological shift was observed: mean Hb declined from 115±18.9 g/L preoperatively to 105±17.9 g/L postoperatively, while moderate anaemia increased from 60 (30.6%) to 99 (50.5%) and severe anaemia doubled from 9 (4.6%) to 19 (9.7%).

Subgroup comparisons revealed that baseline demographics and comorbidities had no significant association with the 30-day composite adverse outcome, including gender X^2^(1, N=196)=0.30, p=0.583, Cramer’s V=0.039 and major comorbidities X^2^(1, N=196)=0.49, p=0.483, Cramer’s V=0.050. Conversely, the operative approach was a highly significant predictor, X^2^(1, N=196)=11.26, p<0.001, Cramer’s V=0.240.

The postoperative anaemia severity model demonstrated superior predictive performance (AUC=0.69; Nagelkerke R²=0.13; AIC=184.10; p=0.001) compared with the preoperative model (AUC=0.67; R²=0.10; AIC=188.10; p=0.005) and the persistent perioperative model (AUC=0.60; R²=0.04; AIC=191.74; p=0.025). A significant dose-response relationship was identified between postoperative anaemia severity and 30-day adverse outcomes: mild anaemia conferred no significant additional risk (OR 1.18; 95% CI 0.45-3.00; p=0.749), whereas moderate anaemia was associated with a 4.3-fold increase in odds (OR 4.31; 95% CI 1.75-10.63; p=0.002) and severe anaemia with a 9.7-fold increase (OR 9.69; 95% CI 1.17-80.41; p=0.035).

Conclusions

Postoperative anaemia severity is a critical, independent predictor of 30-day composite adverse outcomes in vascular surgery, demonstrating a clear dose-dependent risk acceleration at moderate and severe levels. This trajectory is heavily driven by procedural trauma, particularly open interventions. The synergistic impact of an open surgical approach and severe postoperative anaemia dictates poor short-term recovery, highlighting the postoperative period as a vital target for clinical optimisation.

## Introduction

Perioperative anaemia is a frequent finding among patients undergoing major surgical intervention. In the major surgery population, preoperative anaemia is particularly prevalent, affecting an estimated 20-40% of patients, and is established as a potent, independent risk factor for adverse clinical outcomes [1]. In response, standardised strategies for preoperative haemoglobin (Hb) optimisation, such as patient blood management (PBM) pathways, have been widely integrated into clinical practice [2]. However, a significant proportion of patients continue to experience profound postoperative anaemia due to patient comorbidities, the high-risk nature of vascular procedures, and intraoperative blood loss, which preoperative optimisation strategies alone cannot fully mitigate [1].

While preoperative anaemia is a significant concern, postoperative anaemia is ubiquitous, with a prevalence up to 90% following major surgery according to World Health Organisation (WHO) criteria [1,3]. Historically, postoperative anaemia has been dismissed as an inevitable physiologic sequela of surgical trauma rather than a distinct, modifiable clinical condition [2,3]. Consequently, its independent prognostic significance has remained poorly defined compared to its preoperative counterpart. However, emerging evidence suggests that a depleted postoperative Hb mass may drive a cascade of adverse short-term outcomes, including prolonged hospitalisation, increased susceptibility to sepsis, higher readmission rates, and elevated mortality [1,4].

In vascular surgery, both the clinical impact of postoperative anaemia depth and the effectiveness of targeted postoperative management strategies remain insufficiently characterised and limited in the literature, as existing studies have focused predominantly on preoperative optimisation or on general surgical cohorts, leaving this high-risk subspecialty underrepresented in the literature.

Aim and hypothesis

The primary aim was to evaluate the association between the severity of postoperative anaemia and the incidence of short-term adverse outcomes, defined as a composite of prolonged hospital stay (seven or more days), 30-day readmission, and 30-day mortality among adult patients undergoing inpatient vascular surgical procedures at a UK tertiary referral centre.

The secondary aims were twofold: first, to assess the comparative predictive utility of postoperative anaemia severity against preoperative and persistent perioperative anaemia models; and second, to explore whether the strength of these prognostic associations varies across patient gender, composite comorbidities, or surgical approaches (open vs. minimally invasive interventions).

We hypothesised that postoperative anaemia severity would demonstrate a significant dose-dependent relationship with adverse outcomes and provide superior predictive accuracy to preoperative or persistent anaemia models alone.

## Materials and methods

This study was registered as a clinical audit and approved by the Clinical Audit and Effectiveness Team at Nottingham University Hospitals NHS Trust (NUH). Formal ethical approval was not required, in accordance with National Health Service (NHS) Health Research Authority guidance, as the project was defined as a clinical audit. Consequently, public clinical trial registration was not sought, as registry protocols are reserved for prospective interventional research.

Study population

This retrospective, single-centre observational study was conducted at NUH. As the centralised hub for vascular services across Nottingham and Nottinghamshire, NUH operates across the Queen’s Medical Centre and City Hospital campuses, serving a diverse regional population.

Clinical data for all patients undergoing vascular surgical procedures over a four-month period (July 2024 to October 2024) were retrospectively retrieved from electronic health records (EHRs) using relevant clinical procedure codes. Initially, 835 patients were identified (N=835).

Eligibility criteria

Inclusion criteria comprised patients of all ages and genders undergoing any vascular surgical intervention during the study period (July 2024 to October 2024). Exclusion criteria included day-case procedures (e.g. isolated angioplasty with or without stenting), incomplete longitudinal perioperative data (defined as the absence of either a preoperative or postoperative Hb value), and lack of documented 30-day follow-up outcomes.

Briefly, patients of all ages and genders undergoing any vascular surgical intervention during the study period were eligible. To ensure predictive model integrity and maintain an inpatient focus, strict exclusion criteria were applied. No patients were excluded based on the underlying aetiology of their anaemia. The study cohort comprehensively included individuals with all forms of pre-existing or baseline anaemia, encompassing megaloblastic and microcytic types.

The resulting high exclusion rate reflects the retrospective nature of EHR data capture in a mixed elective or emergency vascular service, driven primarily by the exclusion of day-case procedures and cases lacking complete longitudinal perioperative data.

**Figure 1:**
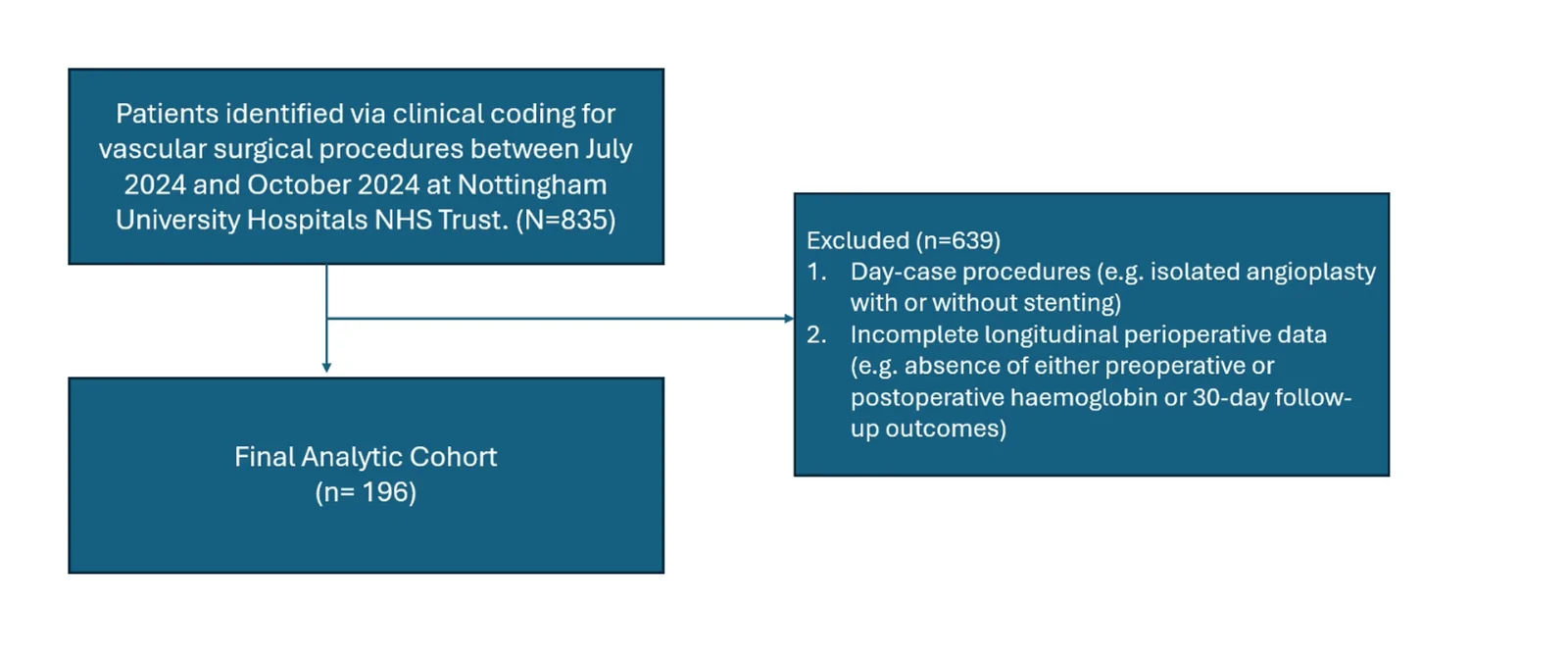
Cohort Flowchart.

Following these exclusions (n=639), a final analytical cohort of 196 patients was established. No missing outcome data were present within the final analytical cohort. The patient selection process and attrition are detailed in the study flowchart (Figure 1).

Study outcomes and variable definitions

The primary outcome was a composite of short-term adverse clinical events occurring within 30 days of the index procedure, including all-cause mortality, unplanned hospital readmission, or a prolonged index hospital stay (seven days and above).

The primary predictor variables were categorised according to the WHO anaemia criteria, utilising sex-specific Hb thresholds [5]. To ensure consistency across models, the same four-level severity scale was applied to both pre-operative and post-operative measurements: no anaemia Hb ≥130 g/L (males) or ≥120 g/L (females), mild anaemia Hb 110-129 g/L (males) or 110-119 g/L (females), moderate anaemia Hb 80-109 g/L, and severe anaemia Hb <80 g/L.

Data collection and sample size

Data were retrospectively collected from the Trust’s digital EHR and Nervecentre, including demographic data (age and gender), comorbidities (diabetes mellitus, hypertension), procedures, laboratory findings (preoperative Hb, postoperative Hb), admission durations, and 30-day follow-up outcomes, which included unexpected readmission and mortality. Data collection was conducted by clinicians following a standardised protocol with predefined clinical procedure codes. The entire extraction process was directly supervised by senior consultant vascular surgeons, and any discrepancies in data categorisation were resolved through consensus compliance reviews.

Due to the retrospective nature of this study, a consecutive convenience sampling strategy was utilised. The cohort comprised all patients who were formally coded for an inpatient vascular surgical procedure within the EHRs during the designated four-month study window (July 2024 to October 2024) at Nottingham University Hospitals. By extracting every consecutive patient matching these specific procedural codes within the index period, selection bias was minimised. A post hoc assessment of sample size adequacy confirmed that the resulting 158 composite adverse outcome events across the final analytical cohort (n=196) significantly exceeded the standard 10 events per variable (EPV) guideline for logistic regression, ensuring robust statistical validity and model reproducibility [6].

Statistical analysis

Data were compiled within Microsoft Excel (Microsoft Corp., Redmond, WA, USA) and analysed using JASP (Version 0.16.0; University of Amsterdam, Amsterdam, Netherlands). A descriptive synthesis of the cohort was performed, with continuous variables summarised as means (±SD) or medians (IQR) and categorical parameters expressed as absolute frequencies and percentages. To delineate clinical differences across various patient profiles, exploratory subgroup analyses were conducted based on gender, procedural classifications (minimally invasive, open surgery), and baseline comorbidities (diabetes, hypertension). For these comparisons, Pearson’s chi-square test or Fisher’s exact test was used as appropriate for categorical data, with statistical significance threshold maintained at p<0.05.

The association between the staged categories of anaemia severity and the primary composite 30-day adverse outcome was initially assessed using bivariate analysis with the chi-square test. Subsequently, binomial logistic regression was used to evaluate three separate predictive models to identify the strongest clinical indicator among the preoperative anaemia severity model, the postoperative anaemia severity model, and the persistent perioperative anaemia model.

The preoperative and postoperative anaemia models were designed to evaluate the association between preoperative and postoperative anaemia severity, respectively, and 30-day adverse outcomes. Anaemia severity was stratified according to the WHO anaemia criteria. The persistent perioperative anaemia model was designed to evaluate the association between sustained anaemia at both the preoperative and postoperative time points and 30-day adverse outcomes. This model was defined as a binary variable (Yes/No), representing patients who met the WHO criteria for anaemia at both the preoperative and postoperative time points.

To identify the optimal prognostic framework, models were appraised for clinical parsimony via the Akaike Information Criterion (AIC) and for explanatory power using Nagelkerke R^2^. The area under the receiver operating characteristic curve (AUC) was calculated for each model to compare their relative discriminative accuracy in predicting 30-day composite adverse outcomes. All metrics were computed directly within JASP's open-source regression framework under standard parametric assumptions, requiring no proprietary software licensure.

## Results

Demographics and comorbidities

A total of 196 patients were identified for the final analysis, and the baseline characteristics were summarised in Table 1.

**Table 1:** Baseline Characteristics and Procedural Data. ^a^ Defined as the presence of either diabetes mellitus, hypertension, or both. ^b^ Percentage based on the total cohort (n=196); subsequent sub-rows are percentages of their respective procedural subgroup.

Characteristics	Frequency (n)	Percentage (%)
Gender (n=196)		
Male	148	75.5
Female	48	24.5
Composite comorbidities^a ^(n=167)		
Diabetes mellitus	105	53.5
Hypertension	129	65.8
Vascular procedures (n=196)		
Open procedures (n=119)	119	60.7^b^
Open amputation	36	30.3
Open endarterectomy	21	17.6
Bypass	25	21.0
Others	37	31.1
Minimally invasive procedures (n=77)	77	39.2^b^
Angioplasty ± stenting	53	68.8
Endovascular aneurysm repair (EVAR)	14	18.2
Others	10	13.0

The cohort was characterised by age profile, with a mean age of 69 years (±15.38). The population was predominantly male, comprising 148 (75.5%) males and 48 (24.5%) females. A high prevalence of metabolic comorbidities was observed, with 167 (85.2%) of patients presenting with composite comorbidities, defined as the presence of diabetes mellitus, hypertension, or both. Individually, hypertension affected 129 (65.8%) of the cohort, while diabetes mellitus was present in 105 (53.5%). The sum of the individual comorbidity frequencies exceeds the composite prevalence due to substantial overlap between the two conditions, with many patients presenting with both hypertension and diabetes mellitus simultaneously.

Surgical procedures

As detailed in Table 1, surgical interventions were categorised according to operative approach. Open procedures accounted for 119 (60.7%) of all operations. Within this subgroup, open amputations were the most common intervention (36, 30.3%), followed by bypass surgery (25, 21.0%) and endarterectomy (21, 17.6%). Other open procedures, including debridement, incision and drainage, and open repair, comprised 37 (31.1%) of the open cases.

Minimally invasive procedures were performed in 77 (39.2%) of the cohort. The majority consisted of angioplasty with or without stenting (53, 68.8%), while endovascular aneurysm repair (EVAR) accounted for 14 (18.2%). An additional 10 (13.0%) of the cases involved miscellaneous endovascular or minimally invasive procedures, including bone biopsy, sclerotherapy, mechanical thrombectomy, and embolisation.

Anaemia prevalence and severity across the perioperative window

**Figure 2:**
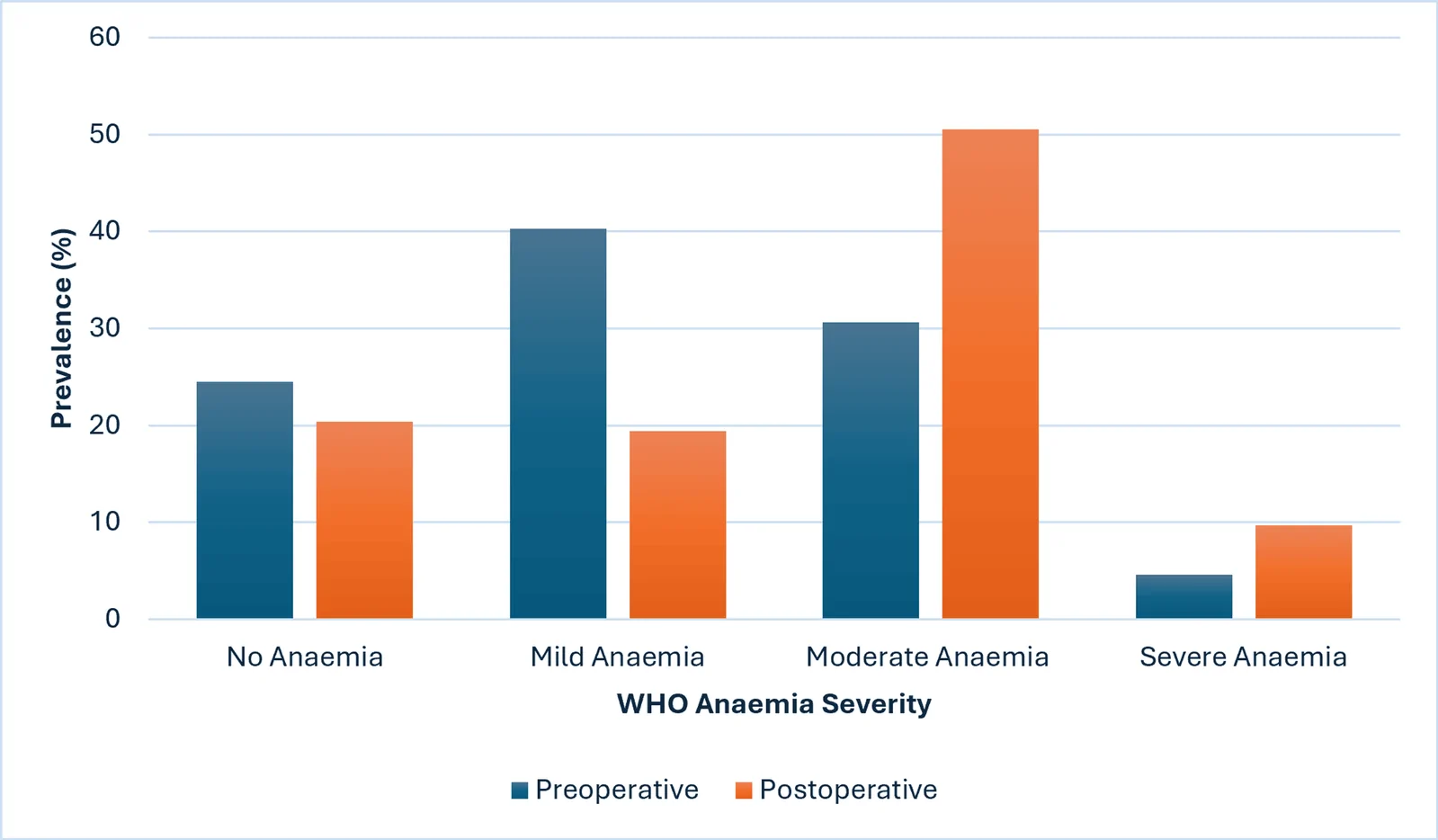
Distribution of Anaemia Prevalence and Severity in the Preoperative and Postoperative Periods (N=196).

The distribution of anaemia severity at preoperative and postoperative time points classified according to WHO criteria is summarised in Table 2 and Figure 2.

**Table 2:** Distribution of Anaemia Prevalence and Severity in the Preoperative and Postoperative Periods (N=196).

Clinical Stages	No Anaemia, n (%)	Mild Anaemia, n (%)	Moderate Anaemia, n (%)	Severe Anaemia, n (%)	Total Anaemia, n (%)
Preoperative	48 (24.5)	79 (40.3)	60 (30.6)	9 (4.6)	148 (75.5)
Postoperative	40 (20.4)	38 (19.4)	99 (50.5)	19 (9.7)	156 (79.6)

Preoperatively, 48 (24.5%) of the cohort presented without anaemia. Within the anaemic population, 79 (40.3%) were classified as mild and 60 (30.6%) were moderate, while only nine (4.6%) presented with severe anaemia.

Following surgical intervention, a significant downward shift in Hb concentrations was observed, with the cohort mean decreasing from a preoperative baseline of 115 ± 18.9 g/L to a postoperative mean Hb of 105 ± 17.9 g/L. This physiological shift was characterised by a distinct migration toward more advanced anaemia categories. While the proportion of patients without anaemia remained relatively stable (40, 20.4%), the prevalence of mild anaemia halved to 38 (19.4%). Conversely, the moderate anaemia cohort increased substantially to 99 (50.5%), and the prevalence of severe anaemia exhibited a two-fold increase, reaching 19 (9.7%) in the postoperative period.

Primary clinical outcomes

The primary composite adverse outcome occurred in 158 (80.6%) patients. Within this group, 140 (71.4%) patients experienced a prolonged hospitalisation (seven or more days), 50 (25.5%) required an unplanned 30-day readmission, and 16 (8.2%) suffered 30-day mortality. As some patients experienced more than one of these complications, the total number of discrete events recorded was 206. A detailed breakdown of these outcomes is provided in Table 3.

**Table 3:** Thirty-Day Adverse Outcomes Following Vascular Surgeries (n=158). ^a ^Percentages of each outcome were calculated based on n=158, and the total percentages of each outcome were >80.6%, as individual patients may have experienced multiple events.

Outcome Measure	Frequency (n)	Percentage (%)^a^
Prolonged Hospitalisation (≥7 Days)	140	71.4
Unplanned 30-Day Readmission	50	25.5
30-Day Mortality	16	8.2
Composite Adverse Outcome	158	80.6

Subgroup analysis and clinical outcomes

Exploratory subgroup comparisons were conducted to evaluate the association of baseline characteristics and the 30-day composite adverse outcome utilising Pearson's chi-square test. The results were summarised in Table 4.

**Table 4:** Chi-Square Analysis of Subgroup Baseline Characteristics and 30-Day Adverse Outcomes.

	Chi-Square Statistic (X^2^)	Degrees of Freedom (df)	p-value	Effect Size (Cramer’s V)
Gender	0.30	1	0.583	0.039
Composite Comorbidities	0.49	1	0.483	0.050
Vascular Procedures	11.26	1	<0.001	0.240

Comparative model performance

The comparative performance of the three evaluated models is summarised in Table 5.

**Table 5:** Comparative Performance for Perioperative Risk Models.

Models	p-value	Akaike Information Criterion (AIC)	Nagelkerke R^2^	Area Under the Receiver Operating Characteristic Curve (AUC)
Preoperative Severity Model	0.005	188.10	0.10	0.67
Postoperative Severity Model	0.001	184.10	0.13	0.69
Persistent Perioperative Model	0.025	191.74	0.04	0.60

The postoperative anaemia model demonstrated the highest predictive accuracy and model fit (AIC=184.10; R^2^=0.13; AUC=0.69). In comparison, lower predictive utility was observed for both the preoperative anaemia model (AIC=188.10; R^2^=0.10; AUC=0.67) and the persistent perioperative anaemia model (AIC=191.74; R^2^=0.04; AUC=0.60).

Association between postoperative anaemia and 30-day adverse outcomes

Analysis of the postoperative anaemia model demonstrated a significant association between the severity of anaemia and the 30-day composite adverse outcome as detailed in Table 6.

**Table 6:** Association Between Postoperative Anaemia Severity and 30-Day Adverse Outcomes.

Postoperative Anaemia Severity	p-value	Odds Ratios (ORs)	95% Confidence Intervals (CIs)
No Anaemia (Reference)	-	1.00	-
Mild Anaemia	0.749	1.18	0.45-3.00
Moderate Anaemia	0.002	4.31	1.75-10.63
Severe Anaemia	0.035	9.69	1.17-80.41

While mild anaemia showed no significant impact on clinical outcomes (p=0.749; OR: 1.18; 95% CI: 0.45-3.00), a strong correlation was observed at higher severity levels. Specifically, moderate anaemia was associated with a four-fold increase in risk (p=0.002; OR: 4.31; 95% CI:1.75-10.63), while severe anaemia resulted in a nearly 10-fold escalation in the odds of an adverse event (p=0.035; OR: 9.69; 95% CI: 1.17-80.41).

## Discussion

Subgroup analysis and clinical outcomes

Baseline demographic characteristics and clinical comorbidities demonstrated no statistically significant association with the composite short-term outcome. Specifically, there was no significant relationship based on patient gender,​​​​​​ X^2^(1, N=196)=0.30, p=0.583, with a negligible effect size (Cramer’s V=0.039). Similarly, the presence of major medical comorbidities, including diabetes mellitus or hypertension or both, failed to demonstrate a significant association with clinical outcomes, X^2^(1, N=196)=0.49, p=0.483, Cramer’s V=0.050.

Conversely, the operative approach was a highly significant predictor of short-term patient outcomes, X^2^(1, N =196)=11.26, p<0.001. This association demonstrated a moderate-to-strong practical effect size, Cramer’s V=0.240, highlighting that the invasiveness of the surgical approach (open versus minimally invasive) is a critical factor in prognostic clinical modelling for this cohort.

A shift in anaemia distribution

This study identifies a notable shift in anaemia distribution, characterised by a migration toward more severe deficiency levels during the perioperative period in vascular surgery patients. Specifically, while 79 (40.3%) of the population presented with mild preoperative anaemia, 99 (50.5%) progressed to moderate postoperative severity and 19 (9.7%) reached severe level. As highlighted by Crispell et al. (2023) and Shah et al. (2023), surgical trauma triggers a significant interleukin-6 (IL-6)-mediated rise in hepcidin [1,7]. This induces a functional iron deficiency by sequestering iron within the reticuloendothelial system, effectively neutralising the patient's ability to mobilise iron stores to compensate for intraoperative losses, thereby explaining the observed migration [1,7].

Comparative model performance and predictive utility

This study demonstrates that postoperative anaemia severity is a potent, independent predictor of 30-day adverse outcomes in vascular surgery patients, with superior discriminative accuracy compared to both preoperative and persistent perioperative anaemia models.

When evaluating predictive accuracy, the postoperative model outperformed both preoperative and persistent anaemia models (AIC=184.10; R^2^=0.13; AUC=0.69; p=0.001). In contrast, we found weaker evidence supporting the relationship between preoperative anaemia (AIC=188.10; R^2^=0.10; AUC=0.67; p=0.005) or persistent perioperative anaemia (AIC=191.74; R^2^=0.04; AUC=0.60; p=0.025) and 30-day outcomes. Our finding aligns with Crispell et al. (2023) and Shah et al. (2023), who posit that the postoperative state serves as a more dynamic indicator of clinical risk [1,7]. This is because it incorporates the cumulative impact of surgical blood loss, haemodilution, and the inflammatory hepcidin blockade, providing a more comprehensive reflection of the patient's physiological status than a static preoperative measure [1,7].

Dose-response relationship between anaemia severity and postoperative outcomes

Our findings demonstrate that while preoperative anaemia is a recognised risk factor in traditional vascular surgery literature, postoperative anaemia severity serves as a significantly more potent indicator of 30-day adverse outcomes. Analysis of the postoperative model identified a clear dose-response relationship, where the risk of the composite 30-day outcome (prolonged hospitalisation seven or more days, unplanned readmission, or mortality) escalated predictably alongside the degree of anaemia severity. Specifically, while mild anaemia did not significantly alter the risk profile (OR: 1.18; 95% CI: 0.45-3.00; p=0.749), the risk escalated sharply for patients with moderate (OR: 4.31; 95% CI: 1.75-10.63; p=0.002) and severe postoperative anaemia (OR: 9.69; 95% CI: 1.17 -80.41; p=0.035), though the wide confidence interval reflects the limited number of patients in the severe anaemia stratum (19, 9.7%) and should be interpreted with caution. Although the present study focuses on vascular surgery, comparable relationships between perioperative anaemia and adverse postoperative outcomes have been reported across other high-risk surgical specialities, including cardiac surgery, suggesting that the adverse impact of perioperative anaemia may represent a broader perioperative phenomenon observed across high-risk surgical populations rather than a finding unique to vascular surgery [4,8,9].

Threshold effects

Our analysis identified a clear severity-response relationship with high odds ratios (ORs) observed in our moderate (OR 4.31) and severe (OR 9.69) cohorts, mirroring the findings of Makar et al. (2024) and Musallam et al. (2011), who demonstrated that the OR of unplanned readmission escalate sharply once discharge Hb falls below 100 g/L [8,9]. This threshold effect is supported by the foundational work of Warner et al. (2023), whose large-scale observational evidence suggests that for every 10 g/L (1 g/dL) decrease in postoperative Hb, the risk of 30-day readmission increases by 8-9% [8,10].

While our study used WHO severity categories rather than continuous Hb values, our "Moderate" and "Severe" groups, both of which fall below the 100 g/L tipping point, captured a similarly significant escalation in risk (OR: 4.31 and 9.69, respectively). This suggests that the WHO Moderate classification serves as a reliable clinical proxy for the high-risk thresholds identified in these large-scale surgical cohorts, confirming that even incremental drops in Hb have a compounded impact on patient recovery.

It should be noted that while Makar et al. (2024) and Warner et al. (2024) focused primarily on readmission rates, our analysis used a composite 30-day outcome. This broader outcome captures the total burden of suboptimal recovery, including both in-hospital delays (prolonged length of stay) and post-discharge complications (unplanned readmission and mortality). This choice of endpoint likely accounts for the high predictive utility of our postoperative model, as it reflects the reality that severe anaemia often results in extended primary hospitalisations, which may statistically mask the risk of subsequent readmission if evaluated in isolation.

The high composite adverse outcome rate (158, 80.6%) warrants careful interpretation, as prolonged hospitalisation (seven or more days) accounted for most events (140, 71.4%). In the context of major vascular surgery, where extended postoperative recovery is frequently required due to procedural complexity and patient comorbidity, a prolonged length of stay may not necessarily indicate significant postoperative morbidity. Consequently, the overall composite adverse outcome rate is likely to be influenced by the inclusion of length of stay as an outcome component and should not be interpreted as indicating that the majority of patients experienced severe postoperative morbidity.

Physiological thresholds in vascular patients

The threshold effect identified in our analysis suggests that while mild anaemia appears relatively well-tolerated, reaching moderate or severe grade represents a critical clinical turning point. The pronounced risk escalation observed in moderate (p=0.002; OR: 4.31; 95% CI: 1.75-10.63) and severe (p=0.035; OR: 9.69; 95% CI: 1.17-80.41) cohorts may be explained by the oxygen supply-demand mismatch described by Leiner et al. (2020) in high-risk surgical patients, whereby haemodynamic instability and impaired oxygen delivery contribute to cellular oxygen debt and postoperative complications [11]. In surgical patients broadly, cardiovascular comorbidity significantly amplifies anaemia-related operative risk; in vascular surgery specifically, coexisting coronary artery disease (CAD) may further reduce tolerance to perioperative haemodynamic stress, with major adverse cardiac event rates reported at approximately 16% following open aortic repair and substantially higher among patients with concomitant anaemia and CAD [12,13]. Once Hb falls below a critical oxygen delivery threshold, tissue oxygen supply dependency ensues, and anaerobic metabolism is initiated, converting moderate haematological deficiency into a primary driver of major clinical morbidity [11,14].

Clinical implications

These findings highlight the postoperative period as a vital window for intervention, particularly for the vascular surgery population. For a vascular patient, moderate anaemia is not merely a laboratory abnormality but a state of haemodynamic fragility that can precipitate myocardial ischaemia or surgical site complications [11]. Therefore, consideration of intravenous iron supplementation in anaemic vascular surgery patients may be warranted in line with current perioperative anaemia guidelines [2]; however, the evidence base for postoperative intravenous iron specifically remains limited, and optimal timing, dosing, and patient selection require further prospective investigation [2,3].

Limitations and future research

Several limitations of this study warrant consideration. Primarily, the single-centre retrospective design and a relatively modest cohort may limit the generalisability of our findings to the extent to which these results can be reliably applied to the broader population of vascular surgery patients in different hospital settings. Within the severe postoperative anaemia stratum, the small sample size (19, 9.7%) contributed to diminished precision and wider confidence intervals for our highest risk estimation. Nevertheless, the monotonic dose-response relationship maintained across all models suggests that the underlying biological association remains statistically coherent, aligning with the Makar et al. (2024) and Warner et al. (2024) studies cited herein [8,15].

Secondly, the absence of serial Hb measurements across the perioperative period prevented the study from distinguishing between acute and chronic anaemia, which may have distinct pathophysiological implications for vascular recovery.

It should be noted that the “Persistent Perioperative Anaemia model” was operationalised as a binary variable (Yes/No) rather than an ordinal severity scale, as the combination of two timepoints did not permit reliable stratification into four severity levels without substantially reducing cell counts. While this approach limits direct comparability of AUC values across models, it retains clinical relevance by capturing the presence or absence of sustained haematological compromise.

Furthermore, the retrospective nature of the data collection meant that data attrition was an unavoidable limitation, as we could not retrospectively recapture lost clinical entries or influence patient selection, which resulted in a high exclusion rate (639, 76.5%), potentially affecting the sample representativeness and introducing selection bias.

This design also introduces the risk of unmeasured confounding, including objective frailty indices, baseline nutritional status, procedural complexity, precise intraoperative blood loss, and the transfusion volumes of whole blood or specific cell components, which fell outside the scope of our data extraction. These factors can independently drive both the depth of perioperative anaemia and the 30-day composite outcome. In particular, the lack of granular data regarding allogeneic red blood cell transfusions is a notable constraint; such interventions not only acutely alter postoperative Hb measurements but also carry independent prognostic weight that can skew 30-day clinical trajectories.

Consequently, while our baseline findings offer valuable foundational insights, they underscore the critical necessity for future well-powered, prospective, multi-centre longitudinal studies. Moving toward a prospective methodology will not only eliminate retrospective data attrition but will also allow for the construction of robust multi-variable risk adjustment models that seamlessly integrate the unmeasured confounders identified in this cohort. Specifically, future protocols should systematically track intraoperative blood loss, blood transfusions, standardised objective frailty indices, and complete baseline iron panels. Furthermore, scaling this research across multiple tertiary centres is essential to increase the sample size within the high-risk severe postoperative anaemia stratum, thereby narrowing our risk estimation confidence intervals. Ultimately, these expanded prospective datasets will be vital to establish definitive, risk-stratified Hb triggers and refine targeted postoperative PBM pathways tailored specifically to the unique haemodynamic requirements of vascular surgery patients.

## Conclusions

This retrospective cohort analysis demonstrates that postoperative anaemia severity is a critical, independent predictor of 30-day adverse outcomes, including prolonged hospitalisation, unplanned readmission and mortality, in patients undergoing vascular surgery. A distinct dose-dependent gradient was identified, with moderate and severe postoperative anaemia precipitating a four- to 10-fold increase in short-term complications. This risk trajectory is heavily driven by procedural physiological stress, as evidenced by the significant association between open surgical interventions and worse outcomes. While preoperative or persistent perioperative anaemia exhibited weaker, less consistent associations, it is the synergistic impact of an invasive open approach and profound postoperative anaemia that dictates a poor patient trajectory. Future prospective, multicentre studies incorporating intraoperative blood loss, patient frailty, and transfusion data are warranted to validate these findings and establish optimal postoperative intervention thresholds.
